# Pharmacokinetics and pharmacodynamics of antibiotics in critically ill acute kidney injury patients

**DOI:** 10.1002/prp2.280

**Published:** 2016-11-24

**Authors:** Welder Zamoner, Fernanda M. de Freitas, Durval S. S. Garms, Mariele Gobo de Oliveira, André L. Balbi, Daniela Ponce

**Affiliations:** ^1^ NephrologyUniversity São Paulo State‐UNESPBotucatuSP18618‐970Brazil

**Keywords:** Acute kidney injury, antibiotics, critically ill patients, dialysis, drug toxicity, sepsis

## Abstract

Sepsis is the most common cause of death in critically ill patients and is associated with multiorgan failure, including acute kidney injury (AKI). This situation can require acute renal support and increase mortality. Therefore, it is essential to administer antimicrobials in doses that achieve adequate serum levels, avoiding both overdosing and drug toxicity as well as underdosing and the risk of antibiotic resistance and higher mortality. Currently, there are no validated guidelines on antibiotic dose adjustments in septic patients with AKI. The current recommendations were extrapolated from studies conducted in noncritical patients with end‐stage chronic kidney disease receiving chronic renal replacement therapy. This study aimed to review and discuss the complexity of this issue, considering several factors related to drug metabolism, the characteristics of critically ill patients, the properties of antimicrobial drugs and dialysis methods.

**Abbreviations**
AKI: acute kidney injury
CRRT: continuous renal replacement therapy
ED: extended dialysis
HD: hemodialysis
HPLC: high‐performance liquid chromatography
IHD: intermittent hemodialysis
MDRD: modified diet in renal diseases
MIC: minimum inhibitory concentration
PD: peritoneal dialysis
PK: pharmacokinetic
RRT: renal replacement therapy

## Introduction

The main cause of death in patients in intensive care units is sepsis, with mortality rates ranging from 18.4 (Kaukonen et al. 2014) to 60%, depending on the severity of the condition (Alberti et al. 2002; Zarjou and Agarwal 2011). In recent years the sepsis, severe sepsis and septic shock concepts have been reviewed and updated targeting more accurate diagnosis and best suitable treatment of this condition. In the last update sepsis was defined as an organic life‐threatening dysfunction caused by exacerbated response to infection (Singer et al. 2016). Sepsis is a well‐known risk factor for the development of acute kidney injury (AKI), taking to 70% mortality rate, greater than other causes of AKI (around 45%; Schier and Wang 2004).

Sepsis is the main cause of AKI in critically ill patients, and half of these patients require acute renal support (Bellomo et al. 2004; Davenport 2011; Zarjou and Agarwal 2011). Thus, the adoption of measures that lead to decreased mortality and costs associated with treatment and hospitalization has become important. Actions with the greatest impact include early administration of antimicrobials, the choice of which is based on the patient's history, the recent use of antibiotics and the source of community or hospital pathogens (Roberts and Lipman 2009).

In a septic patient, variations in the volume of distribution and clearance can affect the antimicrobial concentration. Patients undergoing acute renal support via dialysis also have an increased risk of receiving a subtherapeutic dose of the antimicrobial (Roberts and Lipman 2009; Lewis and Mueller 2014). Maintaining an adequate antimicrobial dose is key to preventing bacterial resistance, infection by opportunistic bacteria and mortality. This is dependent on microbiological activity, antimicrobial sensitivity, and pharmacokinetics (Roberts and Lipman 2009).

To date, there are no validated guidelines on antibiotic dose adjustments in septic patients with AKI; current recommendations have been extrapolated from studies conducted in noncritical patients with end‐stage chronic kidney disease receiving chronic renal replacement therapy (Bellomo et al. 2004; Mueller and Smoyer 2009). This study aimed to review and discuss the complexity of this issue, considering several factors related to drugs metabolism, the characteristics of critically ill patients, the properties of antimicrobial drugs and dialysis methods.

## Pharmacokinetics and Pharmacodynamics of Antibiotics in Critically ill Patients

The antimicrobial exert its effect by different mechanisms, primarily by inhibiting the synthesis of the bacterial wall (penicillins, glycopeptides, carbapenems, and cephalosporins), inhibiting DNA replication (quinolones) or its transcription (rifampicin), impairing bacterial ribosomes and protein synthesis (macrolides, linezolid, dalfopristin, tetracyclines, and aminoglycosides), interfering with metabolic pathways (sulfonamides and trimethoprim) or disrupting the cytoplasmic membrane (polymyxin and daptomycin) (Finberg and Guharoy 2012).

The parameter used to measure the microbiological activity of an antimicrobial is the minimum inhibitory concentration (MIC). This is an in vitro measure of the effectiveness of the antimicrobial against the microorganism (Finberg and Guharoy 2012).

**Figure 1:**
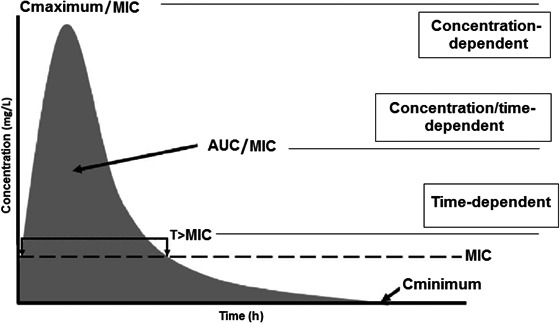
Pharmacodynamics of an antimicrobial drug with respect to its concentration versus time curve. *T* > MIC: time (*T*) that the drug concentration remains above the minimum inhibitory concentration (MIC); Cmaximum/MIC: maximum concentration rate (Cmaximum) by the MIC; AUC/MIC: ratio of the area under the curve (AUC) of the concentration versus time above the MIC. Adapted from Roberts and Lipman (2009).

Pharmacokinetics and pharmacodynamics are tools that determine how much and how often the drug should be dispensed (Finberg and Guharoy 2012). Pharmacokinetics describes the absorption, distribution, metabolism, and elimination of a drug, whereas pharmacodynamics describes the impact of serum levels and the drug response (Roberts and Lipman 2009; Finberg and Guharoy 2012). Thus, the pharmacodynamics of an antimicrobial may be time‐dependent, that is, related to the time of exposure to a specific MIC, such as beta‐lactams, clarithromycin, erythromycin, carbapenems, linezolid, lincosamides (clindamycin) (Finberg and Guharoy 2012), and fluconazol (Fissell 2013); or it may be concentration‐dependent, as for aminoglycosides, metronidazole, daptomicina (Finberg and Guharoy 2012), amphotericin B, and echnocandins (Fissell 2013). The effects of some drugs are both concentration‐ and time‐dependent, as for quinolones, azithromycin, glycopeptides, tetracycline (Roberts and Lipman 2009) (Fig. 1).

To optimize antimicrobial therapy and maximize the effect of the drug on the pathogen, as well as to reduce the risk of antimicrobial resistance and avoid drug toxicity (Blot et al. 2014; Lewis and Mueller 2014), the drug with the correct spectrum of action should be selected, initiated early and given at an appropriate dose based on its pharmacokinetics and pharmacodynamics.

**Figure 2:**
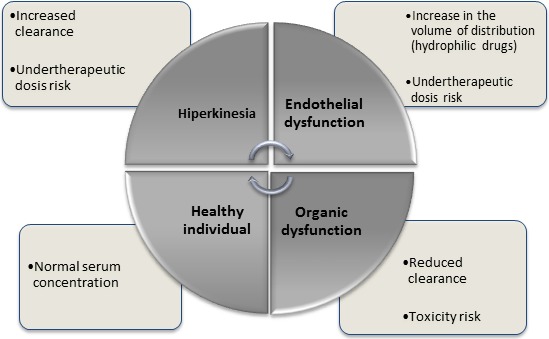
Influence of the patient's clinical status on antimicrobial pharmacokinetics. Adapted from Roberts and Lipman (2009).

Various mechanisms influence antimicrobial pharmacokinetics in critically ill patients (Scoville and Mueller 2013; Blot et al. 2014) (Fig. 2). The absorption of a drug by the oral route of administration may be impaired (Lewis and Mueller 2014) by gastric dysmotility, adherence in circuits, interactions with nutritional components or incorrect gastric pH due to the concomitant use of proton pump inhibitors (Fissell 2013). Similarly, via the subcutaneous route of administration, absorption may be impaired by reduced secondary cutaneous circulation and the redistribution of blood flow exacerbated by edema (Lewis and Mueller 2014). Considering these effects on absorption, preference is given to intravenous administration of antibiotics in critically ill patients.

Antimicrobial distribution also undergoes profound changes in critically ill patients: the production of endotoxins by a microorganism during sepsis can lead to the release of various inflammatory mediators that affect the vascular endothelium and culminate in the altered distribution of blood flow, increased capillary permeability, acid/base disorders and endothelial injury. Thus, fluid extravasation from the intravascular to the interstitial compartment occurs, increasing the distribution volume of hydrophilic drugs and decreasing serum levels; this has been shown with beta‐lactams, aminoglycosides, glycopeptides, linezolid, and colistin. Changes in the volume of distribution can also be increased in the presence of mechanical ventilation, hypoalbuminemia, and extracorporeal circuits (Roberts and Lipman 2009; Roberts 2011; Lewis and Mueller 2014).

Drug elimination also changes under septic conditions; in the absence of organ dysfunction, there is increased renal perfusion and creatinine clearance, leading to increased hydrophilic drug elimination (Roberts and Lipman 2009) and the optimization of other routes of metabolism and elimination (bile and transintestinal routes) (Lewis and Mueller 2014), leading to reduced serum concentrations of some antimicrobials (Roberts and Lipman 2009).

With deterioration of the patient's health status, myocardial depression, and decreased organ perfusion impair the clearance of the antimicrobial (either due to hepatic or renal impairment), increase the half‐life (defined as the time until the concentration of the drug is reduced by half (Roberts and Lipman 2009)) and increase potential toxicity due to elevated serum concentrations of the drug and/or accumulation of its metabolites (Roberts and Lipman 2009). However, patients on acute kidney support show increased drug clearance, depending on the molecular weight, entrainment of proteins and distribution volume (of the drug), and the heterogeneity of the dialysis method, with increased clearance seen using methods that are prolonged, frequent, or more intense (Kielstein and Burkhardt 2011; Lewis and Mueller 2014). Antimicrobial hepatic metabolism may also be affected in the context of AKI. This is not fully understood, but is likely due to by changes in hepatic blood flow and reduced activity of cytochrome P450 enzymes, particularly CYP 3A (Lewis and Mueller 2014).

The standard dose recommendations for antimicrobials were determined in studies performed on healthy young adults and physiologically normal individuals (Roberts 2011). In order to avoid under or overdosing, dose adjustments should be performed in patients with renal or hepatic impairment (Roberts and Lipman 2009; Roberts 2011).

To perform adjustments for renal function, the dosing schedule should be based on the volume of distribution and systemic clearance (Roberts 2011). Estimates of the glomerular filtration rate by indirect methods are not as accurate, despite the ease of monitoring this parameter (Roberts 2011; Blot et al. 2014). Among the available calculations currently used to estimate creatinine clearance, the Cockroft‐Gault equations, the Modified Diet in Renal Diseases (MDRD) score and the Chronic Kidney Disease EPI (CKD EPI) score (Gilbert et al. 2014; Kaukonen et al. 2014) were all validated in patients with stable renal function, which does not occur in the context of AKI (Blot et al. 2014). Thus, the antimicrobial doses currently suggested for critically ill patients with AKI were derived in most cases from studies on patients with chronic kidney disease (Blot et al. 2014).

An alternative method is to measure clearance directly by collecting urine for 24 h or as samples taken every 2, 4, or 8 h using the formula: urinary creatinine concentration × urine volume × time/serum creatinine. The result is expressed in mL/min (Blot et al. 2014); however, this method is impractical and limited in anuric patients.

The therapeutic monitoring of drugs can measure the serum concentration of the antimicrobial, and its clearance can be calculated to improve the accuracy of subsequent dose adjustments, providing a lower risk of toxicity due to overdose and a lower risk of uncontrolled infection or bacterial resistance due to underdosing (Roberts 2011). Among the bioanalytical methods used for therapeutic drug monitoring, immunoassays such as fluorescence polarization (FPIA), multiplied by technical enzyme (EMIT), and immunoenzymatic assays (ELISA) are popular methods using the reaction of an antibody to its antigen. However, drug metabolites or drugs with a similar structure can also be recognized by the antibody, resulting in falsely high concentrations.

High‐performance liquid chromatography (HPLC) and mass net‐spectrometry chromatography (LC/MS) are more specific methods that can separate and quantify drugs based on their molecular polarities and interactions with the stationary phase in a column, but are associated with a high cost and require highly trained technicians, which make these methods difficult to use in medical practice (Liu et al. 2011).

## The Influence of Acute Renal Supportive Therapy

An important factor that interferes with the removal of drugs is the dialysis technique, which may be based on one of two types of transport: diffusion or convection. Both are effective at removing low molecular weight solutes; however, convective therapy is most effective in removing high molecular weight substances.

The choice of the dialyzing membrane also affects drug removal, since a high flux membrane, with increased permeability of medium size molecules, presents a greater capacity to remove drugs with a high molecular weight compared to low flux membranes (Eyler and Mueller 2011; Lewis and Mueller 2014). This difference was demonstrated in a small prospective cohort study (*n* = 9) carried out in the Czech Republic. The study compared the removal of vancomycin in critically ill patients with AKI in hemodialysis with high versus low flow membranes. The median percentage removal of vancomycin after dialysis with a low flow membrane was 17%, whereas with a high flux membrane this was 31%. The study concluded that, despite the differences between removal membranes, it was still necessary to monitor serum levels of vancomycin after each dialysis and to provide an additional dose of vancomycin, since all patients showed subtherapeutic antibiotic levels (Petejova et al. 2012).

Another feature of the dialyzing membrane is adsorption. Hydrophobic synthetic membranes have a high adsorption capacity, whereas cellulose acetate membranes show less adsorption (Clark et al. 1999). The clinical importance of this property of the membrane in relation to interference in serum levels of antimicrobials requires further study, but some evidence suggests early saturation of this process (Schetz 2007).

In critically ill patients, several options for renal replacement therapy (RRT) are available: peritoneal dialysis (PD) and hemodialysis (HD), which can be classified according to their duration and dialysate and blood flow, such as conventional intermittent hemodialysis (IHD), prolonged or extended dialysis (ED), and continuous renal replacement therapy (CRRT) (Pannu et al. 2008; Kielstein et al. 2010). Currently, there is no consensus in the literature as to which is the best method of dialysis for patients with AKI. Thus, the choice of the method made by nephrologists and intensivists, according to their experience and the clinical condition of the patient at the time of treatment (Gabriel et al. 2008). Table 1 shows the types of dialysis and their main features.

**Table 1:** Antimicrobials used in intensive care and their main characteristics (based on The Sanford Guide to Antimicrobial Therapy, reference Gilbert et al. 2014)

	Vancomycin	Meropenem	Cefepime	Piperacillin Tazobactam	Fluconazole	Micafungin
Pharmacodynamics	AUC/MIC	*T* > MIC	*T* > MIC	*T* > MIC	AUC/MIC	AUC/MIC
Molecular weight (Da)	1485	384.46	571.5	539.5 322.3	306.99	1292.26
Volume of distribution^a^ (L/kg)	0.7	0.23–0.35	0.3	0.24–0.4	0.7–0.8	0.39
Protein binding (%)^a^	10–55	2	20	16–48	10	>99

Clearance	Renal	Renal	Renal	Renal	Renal	Hepatic/Renal
Dose for normal renal function	15–20 mg/kg q8–12 h	1 g q8 h	1–2 g q8‐12 h	3.375 g q6 h	100–400 mg q24 h	100–150 mg q24 h
Dose in CRRT	500 mg q24–48 h	500 mg q24 h	2 g q24 h	2.25 g q6 h	200–400 mg q24 h	No dose adjustment
Dose in EHD^b^	No data	No data	No data	No data	No data	No dose adjustment
Dose in IHD^c^	15 mg/kg after HD	500 mg q24 h	1 g q24 h (+1 g after HD)	2.25 g q12 h (+0.75 g after HD)	100–400 mg q24 h – after HD	No dose adjustment
Dose in PD^d^	7.5 mg/kg q2–3 days	500 mg q24 h	1–2 g q48 h	2.25 g q6 h	50–200 mg q24 h	No dose adjustment

AUC, area under the curve; MIC, minimum inhibitory concentration; T, time; CRRT, continuous renal replacement therapy; IHD, conventional intermittent hemodialysis; EHD, prolonged or extended hemodialysis; HDI, intermittent hemodialysis; PD, peritoneal dialysis.

a In healthy individuals.

b Is suggested to be used in the same dosages.

c Considering next IHD in 1 day.

d CAPD (continuous ambulatory peritoneal dialysis).

Peritoneal dialysis is an option for a selected group of patients. Recent studies have suggested that, when indicated, PD should be performed with large volumes of dialysate, in a continuous manner and through a flexible catheter and cycler, in order to obtain survival results similar to patients treated with IHD (Ponce et al. 2012). In PD, the dialyzing membrane is the peritoneum; little is known about drug removal in high volume therapies.

Intermittent hemodialysis is characterized by high blood and dialysate flow, that is, 300–400 and 500 mL/min, respectively, for 4 to 5 h at an affordable cost. This method uses similar machines and filters to those used in chronic dialysis (Fieghen et al. 2010). Intermittent hemodialysis is indicated in hemodynamically stable patients and can be taken on alternate days or daily, according to the clinical and laboratory conditions of the patient, in order to maintain water balance and control the generation of urea (Shingarev et al. 2011).

Some authors suggest that critically ill patients with AKI, as they are hemodynamically unstable (using vasoactive drugs) and hypercatabolic should be treated by continuous methods (Yu et al. 2007). Continuous renal replacement therapy, defined as a prolonged and continuous treatment, lasts for 24 h and uses lower blood and dialysate flow compared to conventional dialysis, that is, 100–150 and 1000–1500 mL/h, respectively (Marshall and Golper 2011). This is an efficient method that provides adequate metabolic and blood volume control without affecting the hemodynamic stability of the patient.

An intermediate method that provides hemodynamic stability and adequate metabolic control of patients with a shorter duration than CRRT is prolonged or ED, lasting between 6 and 18 h. The blood and dialysate flow are lower than in conventional dialysis, that is, 100–200 and 200–300 mL/min, respectively (Kumar et al. 2000; Marshall and Golper 2011).

Intermittent hemodialysis and ED can be performed with low or high capillary flow and efficiency, or with greater or lesser removal means molecules capacity, according to the ultrafiltration and performance coefficients (Kuf and KoA, respectively), the duration of therapy and blood flow variables. Continuous renal replacement therapy is performed using hemofilters (capillaries with a large removal capacity for larger molecules) and low blood flow (Blake and Daugirdas 2008).

Regarding the different dialysis methods, there have been few studies on antibiotic removal in association with DP and ED, and the studies performed on IHD and CRRT were not all done on critically ill patients. So, there are many questions about drug flux in critical patients subjected to the different dialysis modalities.

In clinical practice, the most commonly used guideline is the “Sanford Guide to Antimicrobial Therapy” (Gilbert et al. 2014), which includes CRRT and IHD, and recommends that the dosage of an antimicrobial with ED to be estimated as with CRRT. However, Mushatt et al. (2009) recommend that, for antibiotics administered every 24 h, a supplementary dose should be considered immediately after ED or alternatively, the prescribed daily dose should be given after ED. For drugs administered every 12 h, a dose should be done after ED session and the other after 12 h. Another suggestion is that drugs such as vancomycin and gentamicin, for which serum levels can be measured, should be assessed immediately after ED to determine the need for a further dose after dialysis (Mushatt et al. 2009).

Table 1 shows the pharmacodynamic characteristics (PD) and pharmacokinetic (PK) of the main antibiotics used in clinical practice in intensive care, although the recommended doses have been extrapolated from studies not conducted with the critical AKI population and acute renal support.

## Conclusion

The topics discussed in this review show that the critical patients present several changes in the pharmacokinetics and pharmacodynamics of antibiotics, especially regarding absorption, distribution and metabolism, resulting in variations in serum levels. Thus, there is an increased risk of overdosing and drug toxicity, or a subtherapeutic dose and an increased risk of bacterial resistance, infection by opportunistic germs and mortality.

The removal of antimicrobials by different dialysis therapies in critically ill patients is a complex issue. This depends on the dialyzing membrane characteristics, such as the surface area (efficiency) and size of the pores (flow), as well as drug characteristics, such as water solubility, molecular weight and the extent of protein binding. Moreover, the rate of blood flow, the duration of therapy, and the kind of dialysis (diffusion and/or convection) affect drug removal.

There are no validated guidelines to assist in antibiotic dose adjustment in septic patients on acute renal supportive therapy, and the extrapolated recommendations were obtained from studies on noncritical patients with end‐stage chronic kidney disease receiving substitutive renal therapy. Thus, because of the importance of maintaining therapeutic levels of antimicrobial drugs, more studies on this very complex subject are needed in order to reduce microbial resistance and mortality.

## Disclosures

None declared.
